# Investigating the Impact of LNA-anti-miR-92b, miR-181b, TNF-α, and Piperine on Gene Expression and Cell Viability in Jurkat Cells: Implications for Acute Lymphoblastic Leukemia: LNA-anti- MicroRNAs and Jurkat Cells

**DOI:** 10.31661/gmj.v14i.3566

**Published:** 2025-08-09

**Authors:** Mahmoud Torkamani, Mohammad Mahdi Forghanifard, Vajiheh Zarrinpour, Mani Ramzi, Mahdiyar Iravani Saadi

**Affiliations:** ^1^ Department of Biology, Da.C., Islamic Azad University, Damghan, Iran; ^2^ Hematology Research Center, Shiraz University of Medical Sciences, Shiraz, Iran

**Keywords:** MicroRNA, T cell, ALL, Acute Lymphoblastic Leukemia, Acute Lymphoblastic Lymphoma, LBL

## Abstract

**Background:**

Acute lymphoblastic leukemia/lymphoblastic lymphoma (ALL/LBL), a prevalent pediatric cancer, arises from precursor lymphoid cells and is affected by various risk factors. Abnormal microRNAs (miRs) and dysregulated expression of BCL-2 family proteins significantly contribute to leukemogenesis. Piperine, noted for its anti-tumor capabilities, has demonstrated potential in enhancing the sensitivity of cancer cells to treatment. We aimed in this study to investigate the influence of specific miRs (miR-92b, miR-181b) and TNF-α on the proliferation and viability of the Jurkat cell line, and examined the effects of piperine on miR expression and the genes BAX, BCL-2, and MCL-1.

**Materials and Methods:**

Jurkat T-cells were cultured and treated with LNA-miR inhibitors to selectively suppress miR-181b and miR-92b expression. Cell viability was assessed using the MTT (3-[4,5-dimethylthiazol-2-yl]-2,5-diphenyltetrazolium bromide) assay, while miR-92b, miR-181b, MCL-1, BAX, and BCL-2 mRNA levels were quantified using SYBR Green Real-Time PCR (Polymerase Chain Reaction). SPSS software (version 18) was utilized for statistical analysis.

**Results:**

The study demonstrated effective inhibition of miR-181b and miR-92b through LNA-anti-miR technology. Treatment with LNA-anti-miR-92b and TNF-α reduced Jurkat cell survival, whereas inhibiting miR-181b enhanced viability. BAX expression decreased with LNA-anti-miR-181b and piperine treatment, while BCL-2 expression declined with LNA-anti-miR-92b and piperine treatment. Additionally, piperine treatment increased miR-181b expression while reducing miR-92b and TNF-α expression.

**Conclusion:**

Our findings suggest that inhibiting miR-92b, miR-181b, TNF-α, and BAX using LNA-anti-miR could be a promising strategy for treating ALL. Piperine may enhance this approach by upregulating BAX. Further research is needed to explore these possibilities and develop effective treatments.

## Introduction

ALL/LBL, which stands for acute lymphoblastic leukemia/lymphoblastic lymphoma, encompasses malignancies that arise from precursor lymphoid cells. This term is used because leukemia and lymphoma represent different clinical manifestations of the same underlying disease. ALL is the most frequently diagnosed cancer in pediatrics, with the highest incidence occurring between the ages of two and five. Most cases do not have identifiable environmental or genetic causes [1].

ALL is influenced by a range of risk factors, which can be categorized as maternal and perinatal, genetic, environmental, and socioeconomic factors. Maternal and perinatal factors associated with ALL include use of nitrous oxide anesthesia during childbirth, complications from difficult labor or conditions of the fetus/newborn, uncomplicated physiological jaundice, and the use of supplemental oxygen during birth [2][3]. Genetic factors encompass conditions such as neurofibromatosis type 1, Down syndrome, Bloom syndrome, cleft lip/palate, and ataxia-telangiectasia [4][5][2], as well as specific germline mutations in genes such as PAX5, ETV6, TP53, ARID5B, CDKN2A, and IKZF1[6][7][8][9][10]. Environmental factors linked to ALL include paternal smoking during the pre-conception period. [11], as well as maternal prenatal contact with indoor housing renovation and pesticides [12]. Additionally, socioeconomic factors, including the father’s education level, father’s occupation, and childcare arrangements, have been identified as influential [13]. Advanced paternal age, maternal-fetal loss, increased birth weight, and urban/rural status have also been linked to a higher incidence of ALL/LBL [14][15][16][17].

MicroRNAs (miRs) are a group of small non-coding RNAs that regulate gene expression. They actively participate in many biological processes, encompassing cell growth, apoptosis, and hematopoiesis. Perturbations in miR expression have been closely associated with cancer, manifesting as both upregulation and downregulation of specific miRs, thereby assuming roles as tumor suppressors or oncogenes. Extensive investigations have established compelling links between dysregulated miR expression and hematological malignancies. Consequently, miRs hold tremendous potential as diagnostic markers for cancer, influencing patient prognosis and treatment strategies[18][19][20][21]. miR-92b is a miR that has been implicated in the development and progression of various cancers. It primarily acts as an oncogene, promoting cell proliferation, migration, invasion, and inhibiting apoptosis [22]. One-way miR-92b contributes to tumorigenesis is by targeting and inhibiting tumor suppressor genes such as PTEN [23]. Inhibition of miR-92b has been shown to suppress non-small cell lung cancer cells growth and motility by targeting RECK [24][23]. miR-181b has been shown to play a part in various cancers, including hematological malignancies. It can contribute to tumorigenesis by regulating cell proliferation, apoptosis, and drug resistance. For instance, in certain cancers, miR-181b can promote chemoresistance by downregulating pro-apoptotic genes [25][26]. Multiple anti-apoptotic BCL-2 family members, including BCL-2 and MCL-1, regulate the survival of immune cells. Different subsets of immune cells, such as naive T cells, regulatory T cells, and B cells, have distinct survival requirements dictated by the specific levels of these proteins. Understanding the role of these proteins provides valuable insights into optimal targeting strategies for immunopathology, transplantation rejection, and hematological cancers [27]. Abnormal expression of the BCL-2 gene can disrupt the survivability of B-cell progenitors and potentially affect leukemogenesis. This abnormal gene expression may elucidate the expansion of leukemic lymphoblasts beyond the bone marrow, shedding light on the underlying mechanisms of leukemogenesis [28]. In terms of therapeutic potential, particular combinations of BCL-2 pro-survival proteins, such as MCL-1 plus BCL-XL and MCL-1 plus BCL-2, can be targeted for potential benefits in treating cancers such as melanoma [29]. Additionally, inhibiting solely MCL-1 or in combination with a chemotherapeutic target can be an appealing strategy for inducing apoptosis in BCP-ALL cells, offering a potential therapeutic avenue for this type of leukemia [30]. Moreover, targeting MCL-1 has shown promise as a potent approach for killing myeloma cell lines, suggesting its therapeutic potential in multiple myeloma [31]. BAX and Bak, from the BCL-2 family, act as critical regulators of apoptosis. Upon activation and oligomerization at the mitochondrial outer membrane, BAX and Bak cause permeabilization, a crucial step in initiating apoptosis [32]. By understanding the intricate involvement of anti-apoptotic BCL-2 family members, the aberrant gene expression of BCL-2, and the core regulators BAX and Bak, researchers can uncover novel therapeutic targets and develop strategies for promoting apoptosis in various immunological contexts and hematological malignancies. In cancer therapy, Locked Nucleic Acid (LNA) and Anti-miR strategies have emerged as powerful tools for targeting specific miRs involved in tumorigenesis. LNA has been proposed as safe and effective antisense drugs for cancer treatment through controlling gene expression [33]. Due to the bridge that connects 2′-oxygen to 4′-carbon in the sugar structure, this group of nucleic acid analog is called locked nucleic acid. LNA oligonucleotides have low toxicity and high stability in vivo and in vitro, high transmissibility into mammalian cells, and high solubility in the aqueous phase and increased antisense activity in biological systems [34]. LNA can be used in miR inhibitors, and compete with mRNA in binding to the miR. LNA is transfected to the cell via routine methods, and exhibits sequence-specificity, nontoxic properties, and improved nuclease resistance [35]. For example, LNA-anti-miR-21 has been shown to effectively decrease miR-21 levels in melanoma cells, leading to suppressed cell proliferation and enhanced apoptosis [36]. In pancreatic cancer, LNA-ISH targeting miR-21 has been linked to the prediction of gemcitabine resistance in patients undergoing adjuvant therapy [37]. Similarly, the inhibition of miR-205 using LNA-anti-miR-205 has demonstrated significant anti-proliferative effects in endometrial cancer cells [38]. LNA-anti-miR-221 was shown to effectively decrease cell growth in multiple myeloma cells, helping with the drug resistance. In various malignancies, inhibiting miR-221/222 has led to decreased tumor growth and increased expression of target proteins. Additionally, the inhibition of miR-92a by LNA-anti-miR-92a has demonstrated the ability to reduce miR-92a activity, causing decreased growth and metastasis of endometrial cancer cells [39]. Moreover, the utilization of LNAs targeting the miR-130 family has shown effectiveness in suppressing bladder cancer cell proliferation, migration, and invasion, indicating the therapeutic potential of targeting this miR cluster [40].

Piperine, an alkaloid in black fruits such as Piper nigrum Linn, exhibits antineoplastic effects against different types of cancer cells. It exhibits apoptotic-inducing properties and exerts inhibitory effects on cellular proliferation. Moreover, these compounds have demonstrated their potential to enhance the sensitivity level of cancer cells to treatment [41][42][43][44][45]. To further investigate their role in ALL and cell survival, this study assessed the effects of piperine on the expression levels of miRs, BAX, BCL-2, and MCL-1. We also aimed to explore the specific roles of miR-92b, miR-181b, and TNF-α in the proliferation and viability of Jurkat cells. This research is necessary to potentially identify new therapeutic approaches for ALL, a type of cancer that requires innovative treatment strategies due to its complexity and the need for improved outcomes. By investigating the effects of LNA-anti-miR-92b, miR-181b, TNF-α, and piperine on gene expression and cell viability in Jurkat cells, this study aims to uncover insights that could lead to the development of more effective and targeted therapies for ALL, ultimately benefiting patients by enhancing treatment options and potentially improving survival rates.

## Materials and Methods

**Table:** Table1. The Primers Used in This Study and the PCR Conditions

**Gene**		**Primer sequences (5′-3′)**	**PCR program**
**BAX**	**Forward**	AGCAAACTGGTGCTCAAGGC	95°C/2 min
	**Reverse**	CCACAAAGATGGTCACTGTC	40 cycles
**MCL-1**	**Forward**	TCTCACTTCCGCTTCCTTC	95°C/10 sec
	**Reverse**	CACCTTCTAGGTCCTCTACATG	59.5°C/20 sec
**BCL-2**	**Forward**	GTGGTGGAGGAACTCTTCAG	72°C/15 sec
	**Reverse**	GTTCCACAAAGGCATCCCAG	
**TNF-α**	**Forward**	GGCAAAGTGCTTACAGTGC	95°C/2 min
	**Reverse**	GTGCAGGGTCCGAGGT	40 cycles
**miR-92b**	**Forward**	GTGGTAGGTTGGGATCGGT	95°C/10 sec
	**Reverse**	GTGCAGGGTCCGAGGT	57.5°C/20 sec
**miR-181b**	**Forward**	ACTGACTCCATTCAACGCTGTCG	72°C/15 sec
	**Reverse**	GTGCAATGTCCGAGGT	
**WT1**	**Forward**	CCAGGCTTTGCTGCTGAG	95°C/2 min
	**Reverse**	GTGGCTCCTAAGTTCATCTG	40 cycles
**c-KIT**	**Forward**	TTCTGCTCCTACTGCTTC	95°C/10 sec
	**Reverse**	CTGGATGGATGGATGGTG	59.5°C/20 sec
**CEBPA**	**Forward**	GAAGCACGATCAGTCCAT	72°C/15 sec
		GCCAGATACAAGTGTTGATAT	

### Ethics Approval

All phases of the study received approval from the Research Ethics Committees of Islamic Azad University-Damghan Branch (IR.IAU.DAMGHAN.REC.1403.012).

### Cell Culture

The National Cell Bank of Iran at the Pasteur Institute in Tehran provided the Jurkat human ALL T-cell line. Gibco (UK) supplied the RPMI 1640 medium for the cell culture, to which 10-20% fetal bovine serum (FBS) was added. The medium also contained 100 μg/ml of streptomycin and 100 U/ml of penicillin from Sigma-Aldrich (USA). The culture was carried out in 25 cm² Nunc (Denmark) culture flasks at 37°C in a humidified environment with 5% carbon dioxide. Twice a week, cells were passaged to guarantee steady exponential development.

### Transfection and Treatment

In this study there were three groups, LNA-transfected, scrambled, and control. LNA sequences corresponding to miR-92b’s and miR-181b’s 5′ regions were used to suppress the production of these genes. The website www.mirbase.org provided the miR sequences. Life Technologies (Applied Biosystems, UK) provided LNA-miR inhibitors for miR-92b, miR-181b, and scrambled control oligonucleotides. Jurkat cells (2.5 × 10^5 cells) were sown in a 6-well plate for transfection, and they were allowed to grow to 80% confluence in a day. Next, using the Lipofectamine 2000 reagent (Invitrogen) and serum-free RPMI 1640 medium, the cells were transfected with 50 pmol LNA-anti-miR in accordance with the manufacturer’s instructions and subsequently treated with piperine with a concentrate of 200 μg/ml in serum-free RPMI 1640 medium, according to the manufacturer’s instructions as briefly described earlier [46].

### Measurement of Cell Viability

To assess cell survival following transfection, a detailed protocol was implemented. Seventy-two hours post-transfection, the viability of the transfected cells was evaluated using the MTT assay (3-[4,5-dimethylthiazol-2-yl]-2,5-diphenyltetrazolium bromide) sourced from Sigma, Germany. Specifically, 5 × 10^3 cells were seeded into 96-well plates and incubated for 24 hours at 37°C in a 5% CO2 atmosphere. Subsequently, MTT was added, and the cells were incubated for an additional 4 hours at 37°C, following the manufacturer’s guidelines.

### SYBR Green Real-time PCR

SYBR Green Real-Time PCR was used for the quantitative study of the mRNA expression levels of BAX, MCL-1, BCL-2, miR-92b, miR-181b, and TNF-α. Primers created especially for each miR were used in conjunction with Takara’s (Japan) SYBR® Premix Ex Taq TM II (Tli RNaseH Plus) master mix. For RNA extraction a modified RNX-Plus kit was used. (CinnaGen, Iran). The purity and integrity of RNA were determined by measuring the optical density 260/280 and agarose gel (1.5%) electrophoresis. cDNA was synthesized using PrimeScript RT Reagent Kit (Takara, Japan) According to manufacturer instructions. As directed by the manufacturer, the reactions were performed in an iQ5 thermocycler from BioRad Laboratories (USA). The [2-∆∆Ct] technique was used to quantify the relative expression changes of the miR-92b, miR-181b, TNF-α, BAX, MCL-1, and BCL-2 mRNAs. ∆∆Ct=[∆Ct (treatment) - ∆Ct (non-treatment)] and ∆Ct=[Ct (sample) - Ct (housekeeping gene)]. At least two duplicate wells were used for each real-time PCR [47][48][49][50].All reactions were performed in duplicate. Real-Time PCR reaction program and primer sequences are summarized in Table-1.

### Statistical Analysis

Data analysis was conducted using SPSS (Statistical Package for the Social Sciences) software, version 18. The mean expression levels of miR-92b, miR-181b, TNF-α, BAX, MCL-1, and BCL-2 were compared between treatment and non-treatment using the Student’s t-test. The Pearson correlation test was employed to assess the correlation between the expression levels of miR-92b, miR-181b, TNF-α, BAX, MCL-1, and BCL-2. The expression levels of these genes before and after transfection were compared using the 2-Related-Samples Test. We assessed the normality of the data by examining the skewness and kurtosis values. A P-value of less than 0.05 was considered statistically significant.

### Ethics Approval

All stages were approved by the Ethics Committee of Islamic Azad University-Damghan Branch (IR.IAU.DAMGHAN.REC.1403.012). In this study, human participation follows the ethical standards of the institutional and national research committee and the 1964 Helsinki Declaration and its later amendments.

## Results

**Figure-1:**
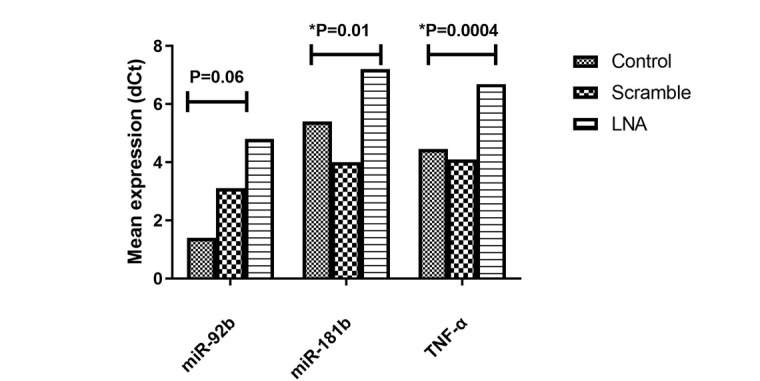
Figure 1. Comparison of gene expression levels in control, scramble, and LNA-transfected samples. Mean±SD dCt values for miR-92b, TNF-α, and miR-181b were compared between LNA and control groups over five experiments. P-values show significance in TNF-α (P=0.0004) and miR-181b (P=0.01) between LNA-anti-miR transfected and control groups.

**Figure-2:**
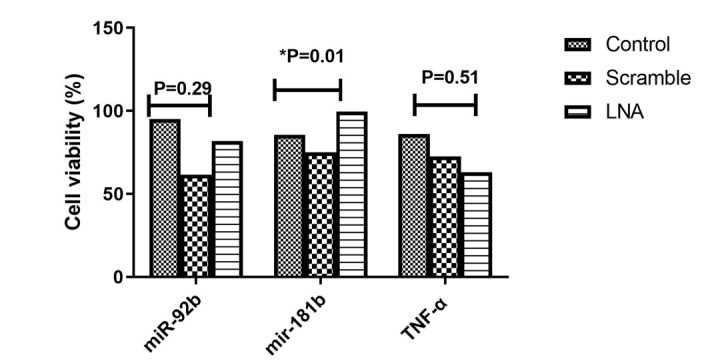
Figure 2. Effect of LNA-anti-miR on miR-92b, TNF-α, and miR-181b blockage on cell viability. Untransfected cells’ viability were considered 100%; other groups (scrambled or LNA) were compared to untransfected using an MTT assay. Data are mean±SD from five experiments. P values show significance in miR-181b(P=0.01) between LNA-anti-miR transfected and control groups.

**Figure-3:**
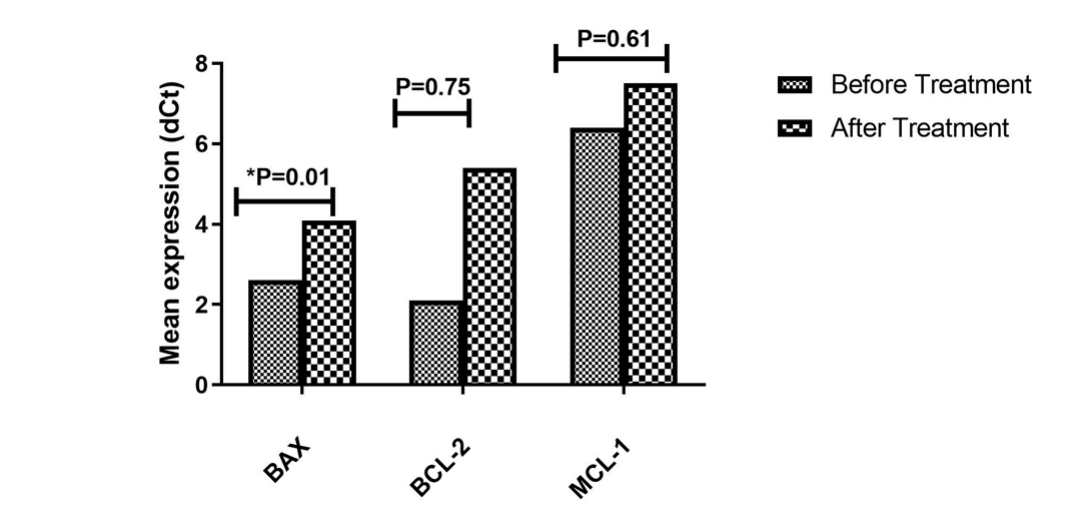
Figure 3. Comparison of gene expression levels before and after treatment for BAX, BCL-2, and MCL-1. Significant increase in BAX expression(P=0.01) observed after treatment. Mean±SD of dCt were compared between transfected and control groups from five experiments.

**Figure-4:**
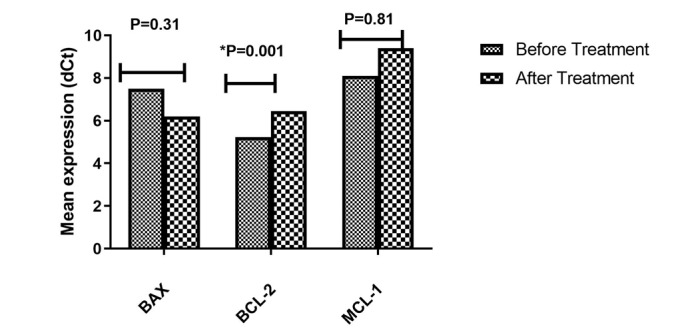
Figure 4. BAX, BCL-2, and MCL-1 expression levels after LNA-anti-miR-92b transfection in Jurkat cells. Mean±SD of dCt were compared between transfected and control groups from five experiments. P-values show significant decrease in BCL-2 expression(P=0.001) after treatment.

**Figure-5:**
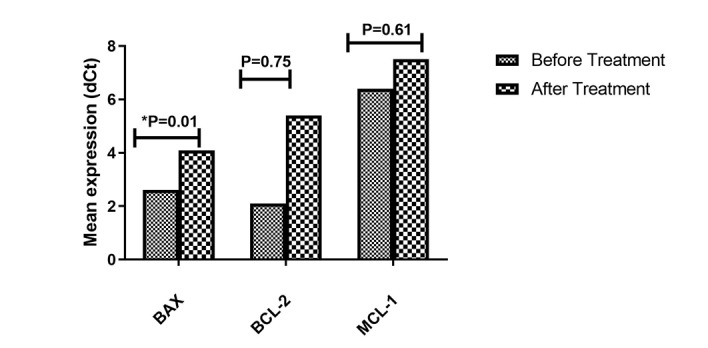
Figure 5. BAX, BCL-2, and MCL-1 expression after Piperine treatment in Jurkat cells. Mean±SD of dCt were compared between treated and control groups from five experiments. P-values show significant increase in BAX expression(P=0.01) after treatment.

**Figure-6:**
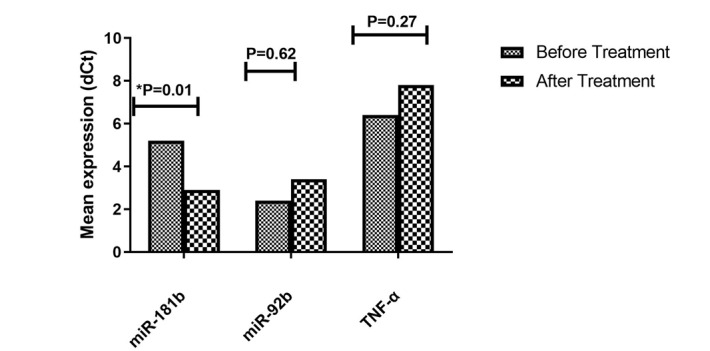
Figure 6. miR-92b, TNF-α, and miR-181b expression changes in Jurkat cells treated with Piperine. Mean±SD of dCt were compared between transfected and control groups from five experiments. P-values show significant decrease in miR-181b expression(P=0.01) after treatment.

### LNA-anti-miR-181b and TNF-α Effectively Inhibit miR-181b and TNF-α Expression

Following transfection, the LNA-anti-miR group showed a substantial decrease in the expression levels of TNF-α and miR-181b in comparison to the control groups (Scrambled LNA or untransfected group) (P=0.01, P=0.0004, respectively). In addition, our results demonstrated that the LNA-anti-miR group had lower miR-92b gene expression than the untransfected or scrambled LNA control groups (Figure-1).

### Blockage of LNA-anti- miR-92b, TNF-α and miR-181b Correlated with Jurkat Cell Line Viability

To assess the impact of blocking LNA-anti-miR-92b, TNF-α, and miR-181b on cell viability, an MTT assay was conducted 72 hours post-transfection. As expected, the viability of the Jurkat cell line was slightly reduced in the scrambled-LNA group compared to the untransfected control group.

However, no significant decrease in cell viability in the LNA-anti-miR-92b and TNF-α transfected groups compared to the control groups at 72 hours post-transfection (P=0.29, P=0.51, respectively) was observed. In contrast, blocking miR-181b significantly increased cell viability (P=0.01, Figure-2).

### Expression of BAX, BCL-2, and MCL-1 in LNA-anti-miR-181b-transfected Jurkat Cells

The findings demonstrated that the transfection of the Jurkat cell line with LNA-anti—miR-181b resulted in a significant decrease (P=0.01) in the mean expression level of BAX between the transfected and non-transfected groups. In contrast, there was no significant change in the mean expression levels of BCL-2 and MCL-1 between the transfected and non-transfected groups (Figure-3).

### Expression of BAX, BCL-2 and MCL-1 in Jurkat Cell Transfected with LNA-anti- miR-92b

The results showed that treatment of the Jurkat cell line with LNA-anti-miR-92b significantly decreased the mean expression level of BCL-2 compared to the untransfected group (P=0.001).

However, the mean expression levels of BAX and MCL-1 did not significantly change in the transfected group compared to the untransfected group (Figure-4).

### Expression of BAX, BCL-2, and MCL-1 in Jurkat Cell Line Treated with Piperine

The results indicated that treating the Jurkat cell line with Piperine significantly reduced the mean expression level of BAX compared to the untreated group (P=0.01). However, the mean expression levels of BCL-2 and MCL-1 remained unchanged in the Piperine-treated group compared to the untreated group (Figure-5).

### Expression of miR-92b, TNF-α, and miR-181b in Jurkat Cell Line Treated with Piperine

The results showed that treatment of Jurkat cell lines with Piperine significantly increased the mean expression level of miR-181b compared to the untreated group (P=0.01). In contrast, the mean expression levels of miR-92b and TNF-α were not significantly different in the treated group compared to the untreated group (Figure-6).

## Discussion

ALL is a common childhood cancer characterized by malignancies of precursor lymphoid cells influenced by various risk factors. Dysregulated miR expression, abnormal expression of the BCL-2 gene, and anti-apoptotic proteins contribute to leukemogenesis [28]. This study examined the effects of LNA-anti-miR-92b, TNF-α, miR-181b, and Piperine on Jurkat cell viability and the expression levels of BCL-2, BAX, and MCL-1.

Firstly, our data revealed that LNA-anti-miR-92b effectively reduced the expression level of miR-92b in Jurkat cells compared to the control groups. This suggests that LNA-anti-miR-92b successfully inhibited the targeted miR. Additionally, the expression levels of miR-181b and TNF-α significantly decreased in the LNA-anti-miR group, indicating the successful suppression of these molecules as well. These findings are coherent with previous studies demonstrating the effectiveness of LNA-anti-miRs in inhibiting specific miR expression [51][52][53].

Furthermore, we noticed a significant decrease in Jurkat cells’ viability that were transfected with LNA-anti-miR-92b and TNF-α in comparison to the control groups. This finding suggests that blocking miR-92b and TNF-α may exert inhibitory effects on cell proliferation or induce apoptosis, which is consistent with previous studies [54][55] that have demonstrated similar outcomes. Conversely, the blockage of miR-181b resulted in increased cell viability, indicating a potential role of miR-181b in promoting cell survival or proliferation. Conversely, the blockage of miR-181b resulted in increased cell viability, indicating a potential role of miR-181b in promoting cell survival or proliferation. These results offer novel insights into anticancer therapeutics by utilizing LNA-anti-miRs, as previously demonstrated in colorectal cancer [56] and melanoma [57]. Furthermore, the targeting and utilization of TNF-α as a therapeutic agent in different breast cancer treatment strategies or soft tissue sarcomas have also been reported [58].

Regarding the levels of BCL-2, BAX, and MCL-1, we observed that the blockage of miR-181b caused a significant decrease in BAX expression, suggesting a potential regulatory role of miR-181b in modulating BAX levels. This finding supports previous research that indicated a positive relationship between miR-181b and BAX expression [59].

However, the levels of MCL-1 and BCL-2 did not significantly change upon miR-181b blockage. These results deviate from previous studies [59][60], which reported an association between miR-181b and alterations in BCL-2 and MCL-1 expression. The inconsistency between our findings and prior research suggests the need for more extensive investigation to clarify the role of miR-181b in BCL-2 and MCL-1 regulation. Similarly, the blockage of miR-92b had no significant impact on BAX and MCL-1 expression levels.

However, it notably led to a significant reduction in the expression level of BCL-2. Other studies have also shown that the BAX/BCL-2 ratio decreased with increased expression of miR-92b-3p, leading to improved cell survival [54].

Furthermore, we explored the effects of Piperine treatment on Jurkat cells. Our results demonstrated a significant decrease in BAX expression upon Piperine treatment, indicating a potential regulatory effect of Piperine on the apoptosis pathway.

However, BCL-2 and MCL-1 expression levels remained unaffected by Piperine treatment. Previous research has also reported the up-regulation of BAX and down-regulation of the BCL-2 gene, highlighting Piperine to be a promising nutraceutical that prevents the progression of CLL [61]and melanoma [62]. Additionally, Piperine treatment led to an elevation in miR-181b expression and a decline in miR-92b and TNF-α expression. Consistent findings were reported in other studies as well [63][64].

While our study provides valuable insights into the role of miR-92b, miR-181b, TNF-α, and Piperine in modulating ALL cell viability and apoptosis, several limitations should be acknowledged. Firstly, our study was conducted in vitro using Jurkat cells, which may not fully recapitulate the complex in vivo microenvironment of ALL.

Future studies should validate our findings in relevant animal models. Secondly, our study focused on a limited number of genes and miRs.

A more comprehensive analysis of the gene expression profile could provide a deeper understanding of the underlying mechanisms. Additionally, further investigation is needed to clarify the inconsistent findings regarding the effects of miR-181b on BCL-2 and MCL-1 expression. Finally, while Piperine showed promising effects in our study, its clinical efficacy and safety in treating ALL require further evaluation in well-designed clinical trials.

## Conclusion

Our findings highlight the critical roles of miR-92b, miR-181b, and TNF-α in ALL leukemogenesis. We found that targeting key regulatory molecules, such as miR-92b, miR-181b, TNF-α, and BAX, using LNA-anti-miR, may offer promising therapeutic avenues. The upregulation of the apoptotic regulator BAX by Piperine is particularly intriguing and warrants further investigation. These results warrant further investigation to explore the combinatorial effects of targeting these pathways and to develop novel therapeutic strategies for ALL.

## Conflict of Interest

The authors have no relevant financial or non-financial interests to disclose.
